# New Technologies in the Workplace: Can Personal and Organizational Variables Affect the Employees’ Intention to Use a Work-Stress Management App?

**DOI:** 10.3390/ijerph18179366

**Published:** 2021-09-05

**Authors:** Giulia Paganin, Silvia Simbula

**Affiliations:** 1 Department of Psychology, University of Milan-Bicocca, 20126 Milano, Italy; silvia.simbula@unimib.it; 2 Bicocca Center for Applied Psychology, Department of Psychology, University of Milan-Bicocca, 20126 Milano, Italy

**Keywords:** smartphone-based interventions, technology acceptance, well-being promotion interventions, new technologies, stress management interventions

## Abstract

Organizations are interested in finding new and more effective ways to promote the well-being of their workers, to help their workers manage work-related stress. New technologies (e.g., smartphones) are cheaper, allow more workers to be reached, and guarantee their anonymity. However, not all employees agree on the use of new technological interventions for the promotion of well-being. Consequently, organizations need to investigate technological acceptance before introducing these tools. By considering the technology acceptance model (TAM) framework, we investigate both the influence of workers’ perceived usefulness and ease of use on their intentions to use apps that help them managing work stress. Moreover, we contribute to the extension of this model by considering both personal (i.e., self-efficacy, personal innovativeness) and organizational (i.e., organizational support for innovation) variables. Our research involved 251 participants who completed an online self-report questionnaire. The results confirm the central hypothesis of the TAM and the influence of other variables that could influence acceptance of new technologies, such as apps that help manage work stress, and the intentions to use them. These results could help organizations ensure technological acceptance and usage by their workers, increasing the effectiveness of new technologies and interventions to promote well-being.

## 1. Introduction

Work-related stress could lead to the onset of several psychophysical conditions (e.g., anxiety, depression, cardiovascular, and gastric disease), leading to an increase in sick days, a higher turnover rate, and loss of productivity and satisfaction [1,2,3,4]. Thus, organization should prioritize interventions to decrease such symptoms [5]. Unfortunately, not all companies have adequate economic/human resources to develop appropriate stress prevention and management interventions. In recent decades, there has been an increase in research studies on alternative ways to deliver such interventions. In particular, there has been a shift from traditional (face-to-face) interventions to technology-mediated interventions [6]. Smartphones, due to their characteristics, are good at delivering these interventions. Smartphones allow continuous, non-intrusive monitoring of participants, reach more people, guarantee their anonymity, and tailor interventions based on individual characteristics and needs [6,7]. Mobile apps that monitor mental health have potential in managing burnout, stress, depression, and anxiety [8,9,10,11]. However, despite some promising results, there is little evidence on smartphone-based interventions [7].

Therefore, before introducing new technologies into the workplace, it is crucial to investigate the acceptance of these new technologies from employees. In particular, the technology acceptance model (TAM) [12] allows us to explore the perceived usefulness/ease of use and the intentions of employees in using these technologies. Subsequent revisions of this model have shown that other external variables (e.g., social norms, facilitating conditions, perceived enjoyment) could help in understanding the acceptance of technology and, in turn, its use [13,14]. 

This study contributes to the research regarding the acceptance of new technology; we investigated the intentions of employees using smartphone apps for stress management. Specifically, we examined the direct and indirect effects of personal (technology-specific self-efficacy and personal innovation) and organizational (organizational support for innovation) variables on the TAM dimensions (perceived usefulness, perceived ease of use, and intention to use). 

The paper is organized as follows: the following paragraphs describe traditional and technology-based well-being promotion and stress management interventions, as well as the technology acceptance model, which represents the theoretical framework of the present study. The second section presents the empirical study. The third section presents the results. The fourth section presents the discussion of the results, considering previous scientific literature and new insight linked to the research context. We conclude with practical implications and limitations of the study. 

### 1.1. Well-Being Promotion and Stress Management Interventions

Work-related stress is typically linked to the onset of psychophysical symptoms, such as depression, anxiety, cardiovascular disorders, gastrointestinal disorders, and sleep disturbances. These disorders, especially when they become chronic, lead to a series of adverse outcomes (for the individual and the organization), such as reduced productivity and performance, increased absenteeism, sick leave, a higher turnover rate, and reduced worker satisfaction and well-being [2,3,15,16,17]. However, the workplace is not only a place where employees encounter risk factors, but also an optimal setting for intervention measures to prevent stress-related health problems and promote health and well-being [17,18,19]. 

Still, research shows that such interventions are often ineffective, or the effect is minimal [7]. For this reason, researchers and companies are searching for new approaches to promote health and well-being at work [7]. New technologies are spreading rapidly in the workplace [20], bringing both benefits (e.g., improved sharing of knowledge, ease of communication) and adverse effects (e.g., information overload, invasion of personal boundaries, technostress) [21,22]. New technologies (regarding health promotion strategies) have allowed researchers, organizations, and professionals to intervene. We have seen a shift from traditional (face-to-face) to technology-mediated interventions (digital interventions) [6]. Workplace use a broad range of digital health interventions (to help manage mental and physical health), including websites, computer programs, smartphone applications, and others [23]. In particular, mobile health (m-health) can be defined as “medical and public health practice supported by mobile devices, such as mobile phones, patient monitoring devices, personal digital assistants (PDAs), and other wireless devices” [24] (p. 6). Interventions that could be delivered and designed using mobile technologies have increased in recent years [25]. The smartphone allows researchers to integrate stress management interventions in individuals’ daily lives, providing unobtrusive monitoring of their activities, delivering interventions at the right moment [26].

Furthermore, smartphone-based applications may help motivate people to adopt lifestyle changes and healthy behaviors [18]. Web and mobile apps for monitoring mental health have displayed potential for managing burnout, stress, anxiety management, and education [21,22,23,24]. However, there is little evidence on digital mental health interventions in the workplace [7,27]. The literature underlines some barriers to the effective implementation and effectiveness of mobile-based interventions in the workplace, such as a high turnover rate due to low employees’ engagement with these interventions [28,29]. 

One strategy to prevent non-participation from employees in digital mental health interventions, specifically programs that utilize new technologies via smartphone apps, is to investigate the employee acceptance of such technologies. It, therefore, appears crucial to understand that technology acceptance is necessary for the effective implementation of technologies in the organization. 

### 1.2. Technology Acceptance Model

The technology acceptance model was previously developed [12] to understand the underlying reason for using technologies. The TAM is based on a consolidated theoretical model, explicitly inspired by the theory of reasoned action (TRA) [30,31]. The TAM is composed of three principal dimensions: perceived usefulness (PU), perceived ease of use (PEOU), and behavioral intention to use (INT).

PU is defined as the belief that using technology may bring advantages and benefits. On the other hand, PEOU is conceptualized by the belief that such technology is easy to use and requires minimal effort. Finally, INT is defined as the likelihood that a person will use such technology [32]. Originally, TAM included attitude as an antecedent of INT, but it was eliminated in order to lighten the model [33,34]. In summary, the model states that PU and PEOU are the perceptions that can result in acceptance. Furthermore, the TAM states that ease of use can influence the perception of usefulness. This is because, given the same features and functions, the easier a technology is to use, the more useful it will be perceived [35]. The TAM has been widely used as a theoretical model for user acceptance in different contexts/types of technologies [36,37,38]. In particular, it is considered a parsimonious theoretical model that explains informational technology adoption by users [38,39]. However, it has been modified to consider external factors that could influence the user’s acceptance in several work contexts [38]. Indeed, some theoretical extensions have been developed (e.g., TAM2 [40], TAM3 [41], UTAUT [42], UTAUT 2 [43]). A common aspect of these revisions relies on the consideration that other variables (i.e., personal and organizational features), besides PU and PEOU, may influence the acceptance and intention to use a given technology [44], even if PU seems to be one of the most relevant predictors of INT [12].

### 1.3. Different Ways to Perceive: The Influence of Individual and Contextual Resources

We should note that both perception of usefulness and ease of use are not simply shaped by usefulness and ease, per se, but are also determined by the user’s psychological traits [45]. Individual differences concern the dissimilarities among people, including perceptions, behaviors, personality traits, and characteristics [46]. Such individual differences are crucial when studying the variables that influence one’s acceptance of new technology [47]. However, it is not clear enough how these individual differences influence acceptance in the work context, given the companies’ difficulty to manage such differences in this field. A previous study [45] showed that, among the variables that could be taken into account, self-efficacy and personal innovation are particularly influential in accepting technology. Furthermore, gender and age may influence the determinant of technology acceptance [48].

#### 1.3.1. Individual Differences

General self-efficacy is defined as a person’s belief in his or her capacity to complete a given task [49]. By considering the technology field, computer self-efficacy refers to ‘the judgment of one’s capability to use a computer’ [50] (p. 192). This type of technology-specific self-efficacy has been adapted to different contexts, such as smartphones [51,52]. Previous studies have confirmed the positive relationship between technology-specific self-efficacy and TAM dimensions [37,53,54,55,56,57]. The results illustrate that technology-specific self-efficacy has an important effect, both direct and indirect, on the intention to use the system. In particular, through the PEOU mediating effect, computer self-efficacy substantially affects the intention to use technology [37]. Another study by [57] showed a direct impact of computer self-efficacy on student intentions to use a virtual classroom. For this reason, the self-efficacy variable should be included for better comprehension of technology acceptance. However, despite such evidence, some studies, i.e., [56,58], found that, contrary to their assumption, computer self-efficacy did not influence the perceived ease of use. These conflicting results led us to believe that the role of technology-specific self-efficacy needs further investigation. 

On the other hand, personal innovativeness in information technology (PIIT) is a personality trait that expresses ‘an individual’s willingness to try any new computer technology’ [47] (p. 206). It represents a different concept from a more global formulation of the construct of innovativeness [59]. The degree of personal innovativeness, both in general and in its declination towards technology, seems to be robustly related to TAM constructs. Indeed, several studies related this variable to intention to use [60,61]. A recent paper by [62] showed that personal innovation directly impacts intention to use. Their results are in line with what has been theorized by [47] and [63].

#### 1.3.2. Organizational Variables

Considering only individual variables in technology acceptance could result in neglecting various elements (e.g., organizational factors), which could facilitate or hinder individual attitudes towards innovation. Indeed, the literature states that employee innovation adoption could also rely on organizational aspects [38,64,65,66]. In particular, the idea that some organizational factors can foster technology acceptance was already considered in the TAM model’s first formulations and finds support in other studies, also conducted through qualitative methodologies [32,66,67,68]. Therefore, it is necessary to consider such factors to ensure the effective introduction of change in the company [66]. For example, organizational support for innovation (OSI), namely ‘the expectation, approval, and practical support of attempts to introduce new and improved ways of doing things in the work environment’ [69] (p. 28), seems to be a key factor that can facilitate the adoption of new technologies [38]. Indeed, some studies found significant relationships among OSI and the usefulness/ease of use of technology [69,70,71,72], as well as intention to use [73]. On the contrary, a lack of organizational support can undermine the company’s efforts to introduce new technology [38,74]. Moreover, a study on technology-enhanced learning underlined the responsibility from managers to offer support and the intention to use technology through perceived ease of use and usefulness. 

### 1.4. Study Hypotheses

Based on the consideration presented above, we aimed to confirm the validity of the TAM within a sample of workers in northern Italy, regarding the intention to use an app that promotes well-being and work-related stress management. Furthermore, we investigated the factors that may influence the perception of ease and usefulness of this app: specifically, we considered both individual factors (i.e., smartphone self-efficacy and personal innovation for technology) and contextual factors, such as organizational support for innovation. The hypotheses of the study are as follows:

**Hypothesis** **H1.** *Perceived usefulness (a) and perceived ease of use (b) are significantly associated with the intention to use a smartphone app for work-related stress management. Moreover, perceived ease of use (c) will directly impact perceived usefulness.*

**Hypothesis** **H2.** *Personal innovativeness with technology is significantly associated with the intention to use a smartphone app for work-related stress management.*

**Hypothesis** **H3.** *Smartphone self-efficacy is significantly associated with the intention to use a smartphone app for work-related stress management, directly (a) and indirectly, via perceived ease of use (b) and through perceived ease of use and perceived usefulness, in series (c).*

**Hypothesis** **H4.** *Organizational support for innovation is significantly associated with the intention to use a smartphone app for work-related stress management, directly (a) and indirectly via perceived ease of use (b), via perceived usefulness (c), and through perceived ease of use and perceived usefulness, in series (d).*

**Figure 1:**
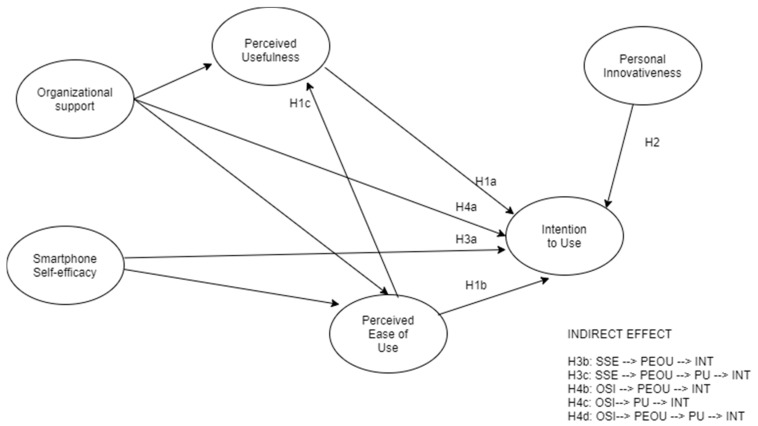
Hypothesized model. Note. SSE = smartphone self-efficacy; PEOU = perceived ease of use; INT = intention to use; PU = perceived usefulness; OSI = organizational support for innovation.

These hypotheses gave rise to the structural model depicted in Figure 1.

## 2. Materials and Methods

### 2.1. Participants and Procedure

Data collection was carried out in July 2020, at three Italian companies in northern Italy. All participants were white-collar workers. 

Each HR department sent an e-mail to the employees to inform them about the possibility of participating in this study. This e-mail specified that participation in the research was voluntary; the University of Milan-Bicocca entirely managed the study, and that the company would not possess the answers given by the workers. After eight days, a second e-mail was sent to workers, reminding them about the possibility of participating in the study.

Before filling in the questionnaire, participants had to read the informed consent documents and indicate whether they consented to participate in the research voluntarily. Informed consent allowed participants to gather information about the study’s objectives and the data collection procedure, and it guaranteed that there would be no associated risks or costs. The research team ensured participants that their data would be used in an aggregated, non-individual manner, to ensure the confidentiality and anonymity. The data collection was conducted in conformity with the ethical standards established by the Declaration of Helsinki and was authorized by the Ethical Committee of the University of Milano-Bicocca (Prot. N. RM-2020-312). Finally, the researchers’ contact details were provided in order to allow participants to contact them to clear up any concerns. In total, 251 responses (11.20% response rate) were included in our dataset. Among them, 61% were female, and 39% male; the mean age was 39.89 years (SD = 9.45).

A total of 20.7% have a high school diploma; 54.2% have a bachelor’s or master’s degree; 23.1% have a higher educational level, and only 2% have a different or lower study level. The mean level of seniority on the job was 15.19 years (SD = 9.54). Regarding health status, 6.4% reported suffering from chronic psychophysical health conditions (e.g., panic attacks, psychosomatic symptoms, chronic headaches, etc.). Concerning previous experience with smartphones, almost all participants (99.4%) were familiar with using a smartphone, and 15.4% had used a stress management or well-being promotion app in the past. 

A detailed description of an existing app was presented to ensure that the workers understood the definition of a smartphone app for well-being and stress management, without applying any exclusion criteria concerning previous experience with such apps. This description also encompassed some images, as well as a text explanation of app functionalities. This app provided several functions: daily mood tracking, numerous courses with expert figures who introduced the users to the world of meditation and different thought processing techniques, access to various coping strategies to manage stressful situations, and weekly monitoring of stress levels, depression, anxiety, and resilience through objective and clinically validated scores. The app also quantified user progress over time and provided a community to share personal stories and chat with other users, to share moods and thoughts.

### 2.2. Measures

The TAM dimension of perceived ease of use was assessed through three items (e.g., “It will be easy to use the app”), perceived usefulness was measured with four items (e.g., “The presented app could help me improve my work-related well-being”), and intention to use was measured through two items (e.g., “I would like to try the presented app”). The items were assessed through a Likert scale from 1 = strongly disagree to 5 = strongly agree. The items were all taken from previous studies [32,75]. Personal innovativeness with technology was assessed via the personal innovativeness with informational technologies (PIIT) four-item scale [76] (e.g., “If I heard about new information technology, I would look for ways to experiment with it”). Respondents were asked to indicate how strong they disagree or agree with the presented statement using a seven-point scale (1 = strongly disagree; 7 = strongly agree).

Computer self-efficacy was evaluated through eight items taken from the computer self-efficacy scale (CSES) [50], assessed on a 10-point scale (1 = not at all confident; 10 = totally confident). The scale was adapted to smartphones, the technology used in our research (e.g., “I could utilize a smartphone app as the one presented... if I had seen someone else using it before trying it myself”). 

TAM measures, PIIT, and CSE items were all translated into Italian using back-translation techniques [77].

Organizational support for innovation was investigated through an eight-item scale [78] (e.g., “This organization is open and responsive to change”), validated in Italian [79]. Responses were on a five-point scale from 1 = strongly disagree to 5 = strongly agree.

### 2.3. Data Analysis

We initially performed descriptive statistics, Pearson correlations, and Cronbach’s alpha coefficients using SPSS 27 (IBM, Armonk, NY, USA). The objective was to observe the sample characteristics, the correlations between variables, and the scale reliability. To test our hypotheses, we performed a full structural equation model (SEM) using Mplus version 7 (Muthén & Muthén, Los Angeles, CA, USA). The model included both the simultaneous indirect effect of the organizational support and the perception of specific self-efficacy on the intention to use a smartphone app, via the perception of its ease of use and usefulness, and the direct effect of personal innovation on the intention to use it. Moreover, we added the effect of gender and age on the technology acceptance variables. Gender and age were intensively investigated as some of the main demographic variables related to ICT adoption [48], although not included in the original formulation of the TAM [80,81]. For example, some studies underline that effort expectation (perceived ease of use) is the strongest predictor for women, and performance expectation (perceived usefulness) is the strongest predictor for men [82]. Older people tend to be change-resistant; thus it is more challenging for them to learn and use [83]. However, past studies on the effect of gender and age on behavioral intention, in various contexts and applications, have shown mixed results [84] that need further investigation. Maximum likelihood was employed as the estimation method. To assess the model goodness-of-fit, we used the statistic criteria listed below: the non-significant χ^2^ value (this statistic suggests that if χ^2^ is non-significant, the model fits the data); root mean squared error of approximation (RMSEA; values smaller than 0.08 indicated an acceptable fit); the comparative fit index (CFI) and the Tucker–Lewis index (TLI; values between 0.90 and 0.95 showed an acceptable fit); the standardized root mean square residual (SRMR; values smaller than 0.08 indicated a proper fit). Through bootstrapping procedures, it is possible to perform repeated subsample simulations from an original dataset. For this reason, the bootstrapping method was conducted to assess the significance of the hypothesized indirect effects.

## 3. Results

### 3.1. Descriptive Statistics

Study variables included standard deviations, correlations, and Cronbach’s alpha values; presented in Table 1. 

All significant relationships between the variables were in the direction suggested by the previous literature. 

In particular, intention to use positively correlated with perceived usefulness (r = 0.722 **, *p* < 0.01) and perceived ease of use (r = 0.502 **, *p* < 0.01). Moreover, age was negatively and significantly correlated with perceived ease of use (age: r = −0.130, *p* < 0.05) and with the intention to use (r = −0.140, *p* < 0.05). On the other hand, gender did not show any significant correlation with the TAM variables. Except for the dimensions of the technology acceptance model, it should be noted that the values of the correlation coefficients varied from moderate (|0.30| < r< |0.49|) to low (r < |0.29|), indicating, respectively, medium and low correlations among variables [85]. All scales presented good reliability, assessed via Cronbach’s alpha: the values were higher than 0.80, the generally accepted standard [86] (see Table 1).

### 3.2. SEM Analyses

**Figure 2:**
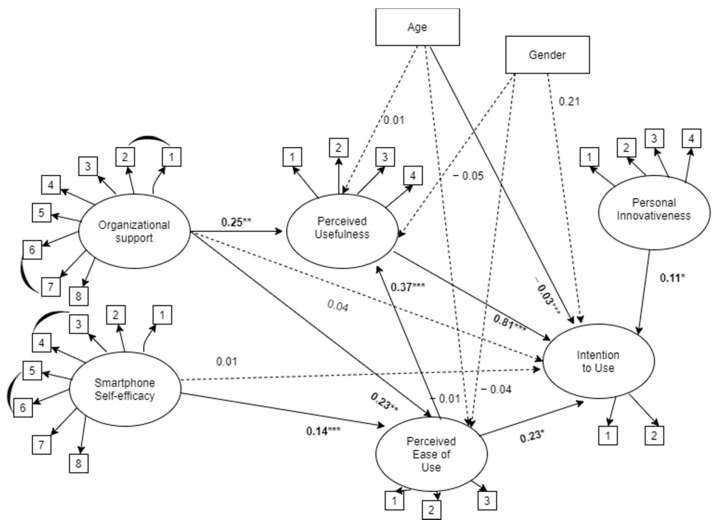
Structural equation model direct effects. Note. * *p* < 0.05; ** *p* < 0.01; *** *p* < 0.001. Straight arrows indicate significant direct effects; dotted arrows indicate non-significant direct effects; curved arrows indicate error correlations. Ovals indicate latent variables; squares indicate observed variables.

The hypothesized model (M1) fit was barely adequate (χ^2^ (417) 819.659, *p* < 0.00, CFI = 0.93, TLI = 0.92, RMSEA = 0.06 (90% CI 0.06, 0.07), SRMR = 0.06. After M1 modification indices review, the following model, (M2), depicted in Figure 2, showed an increase in model fit (χ^2^ (413) = 717.63, *p* < 0.001, CFI = 0.95, TLI = 0.94, RMSEA = 0.05 (90% CI 0.05, 0.06), SRMR = 0.05. Specifically, correlations were added between errors items of the self-efficacy (CSES5 with CSES6; CSES4 with CSES3) and organizational support scales (OSI1 with OSI2; OSI7 with OSI6). 

After checking the goodness of fit indices of the models, we compared the two models through the delta chi-squared test. The results are indicated in Table 2 and point to model 2 as the final model. Consistently, M2 showed well-defined factor loading for all of the observed variables. The model accounted for 49% (R^2^ = 0.49) of the variance in intention to use. Moreover, the model accounted for 24% (R^2^ = 0.24) and 20% (R^2^ = 0.20) of the variance, respectively, in perceived usefulness and perceived ease of use.

Our model results, coherent with H1, confirmed the direct effect of perceived usefulness (H1a) and perceived ease of use (H1b) on intention to use, and the direct effect of perceived ease of use on perceived usefulness (H1c). 

Our results supported the direct effect of personal innovativeness with technology on intention to use (H2), but they did not support the direct effect of smartphone self-efficacy (H3a) and organizational support (H4a) on intention to use. However, the results supported H3b that is, the indirect effect of smartphone self-efficacy on intention to use went through perceived ease of use (β = 0.05, *p* < 0.043) and confirmed the indirect effect of smartphone self-efficacy on intention to use via perceived ease of use and perceived usefulness, in series (H3c; β = 0.07, *p* < 0.001).

Moreover, we confirmed the indirect effect of organizational support on intention to use through perceived usefulness (H4c; β = 0.120, *p* < 0.004), and through perceived ease of use and usefulness, in series (H4d; β = 0.04, *p* < 0. 009), while the indirect effect of organizational support on intention to use via perceived ease of use was not significant (H4b; β = 0.03, *p* < 0. 074). Finally, by considering gender and age, only the latter showed a significant negative effect on the intention to use (β = −0.03, *p* < 0. 000). Table 3 shows all the indirect effects assessed via the bootstrapping method. In Figure 2, we present the direct effects.

## 4. Discussion

This study aimed to deepen our knowledge of which factors may affect acceptance of new technologies; it advises on how to promote innovation implementation and the effectiveness of digital prevention and promotion projects. Indeed, understanding user perceptions towards the intention of using new technology, such as a smartphone app for stress management and well-being promotion, could facilitate implementation and participation in such digital interventions. 

Our study results show that both personal and contextual variables influence one’s intention to use a smartphone app for stress management and well-being promotion, providing valuable insight into the role of personal innovativeness with technology, smartphone self-efficacy, and perceived organizational support for innovation.

Our first hypothesis concerns the impact of the dimensions of perceived ease of use (H1a) and perceived usefulness (H1b) on one’s intention to use a smartphone app for stress management and well-being promotion and the direct relationship of perceived ease on perceived usefulness (H1c). The study results confirm our hypothesis, in line with the theories and findings of previous studies [12,35,48,66]. Indeed, as explained by the TAM, both the perception of ease of use and usefulness directly impact the intention to use. It is interesting to note that, in our results, and similar to the first Davis studies [12,32], the perception of usefulness has a more significant impact on intention to use than ease of use, although the latter also has a considerable influence. This suggests that it could be beneficial for an organization to underline the advantages of new technology, clarifying the benefits for employers. This assumption is valid, both when the proposed technology is intended to support workers in performing their work tasks, and when the introduced technology may be used as a tool to deliver interventions, for the prevention and promotion of employees’ health and well-being.

Regarding the antecedent role, i.e., ease of use on perceived usefulness (H1c), our results align with those shown by others [12,35]. We can hypothesize that the easier a tool is perceived, the more users imagine they have more time and resources to achieve their goals, consequently increasing the perceived usefulness of such a tool. This can be explained by imagining that users are driven to use a tool (in this case, a smartphone app) because of its usefulness (i.e., the possibility of one managing work-related stress and improving well-being), and secondly, by its ease of use. Hence, it is important that the app designer (or the technology designer) also consider the ease of use of the technology, particularly if it is to be introduced in an organizational context and for performing specific tasks. 

In order to not neglect personal factors that could influence the perception of ease of use, usefulness, and the intention to use the smartphone app, we investigated the effect of some personal variables, such as personal innovation (H2) and specific self-efficacy (H3), which were shown in the literature to have a significant impact on the TAM dimensions [45]. 

Therefore, our second hypothesis refers to the direct impact of personal innovativeness with technology, using a smartphone app for stress management and well-being promotion (H2). Personal innovation with technology (i.e., an individual’s willingness to try new technology) [47] appears to be a key personal variable in explaining the intention to use new technology. The study results confirm our hypothesis and are in line with previous literature. Indeed, previous results [60,62,86] indicate that personal innovativeness might increase one’s intention to use a new technological tool. The reason might be that personal innovation is linked to the desire to learn how to use new tools and to experiment. In our case, participants with a higher level of innovativeness could be prompted to try a new technological tool (i.e., a smartphone app) to manage their stress levels and manage their well-being. 

Our third hypothesis is related to the direct effect of smartphone self-efficacy on intention to use (H3a). Contrary to the results showed by [57], we do not confirm the direct effect of self-efficacy on intention to use. However, we show the indirect effect of smartphone self-efficacy on intention to use through perceived ease of use (H3b), the serial mediation of perceived ease of use, and perceived usefulness (H3c). Our results may indicate that it is not enough for workers to feel competent in using a smartphone to be willing to use an app for promoting well-being and managing stress. Instead, our findings underline that the more individuals perceive themselves as capable of using a technological tool, the less effort they expect will be required to use it.

Moreover, as mentioned above [12,35], the less effort individuals estimate they will have to invest, the more energy they will save and, thus, use to achieve their goals. In our present scenario, participants who perceived themselves as more self-efficacious in using smartphones also perceived smartphone stress management apps as easier to use and, consequently, they were more likely to use it (H3b). This is confirmed by previous studies e.g., [37]. Additionally, as they perceived the apps as easier to use, they also perceived them as more useful; thus, increasing their intentions to use them if made available (H3c). 

Our last hypothesis involves the relationship between perceived organizational support for innovation and the intention to use the smartphone app for stress management. In performing our analyses, we hypothesized a direct effect of organizational support on intention to use (H4a), the role of the mediator of perceived usefulness (H4b) and perceived ease of use (H4c), and the serial mediation of perceived ease of use and usefulness (H4d). The results of our analysis only confirm H4b and H4d, but do not confirm the direct relation between perceived organizational support and intention to use (H4a) and via perceived easiness of use (H4c). Therefore, only the simple indirect effect through perceived usefulness and the serial mediation via perceived ease of use and perceived usefulness were found. In particular, as previously stated by [12], perceived ease of use enhanced the perceived usefulness, which in turn increased the likelihood that people intended to use such technology. We can assume that, as stated by [12], perceived usefulness is more influential on intention to use than perceived ease of use, including in the smartphone-based app context. However, smartphone apps are well known by people, and the ease of use, in terms of simplicity and time saving, influences the perceived usefulness of the app in terms of goals that can be achieved, which in turn impact the worker’s intention to use. Combined, our results suggest that workers are interested in the technical and practical aspects of technology and their organization’s contextual and supportive features. This shows how significant it is for organizations to support innovations in general, and to support requests for help in using technology, in order to enhance the perception of usefulness and ease of use of such tools and, subsequently, the employees’ intentions to use them. 

As mentioned above, gender and age are considered key determinants of technology acceptance, as shown by various studies [48,83]. However, our results only show a negative and significant direct effect between age and intention. These results are in line with previous literature that underlined the role of age on intention to use new technology. Previous studies have shown that age impacts the perceived ease of use of new technologies and the perception of its usefulness. In particular, a study on the acceptance of virtual reality [87] stressed that, for younger people, the perceived usefulness of technology is more relevant.

In contrast, for older people, who believe they have less technological skills, the perception of ease of use had a more significant influence on their intention to use and adopt this technology. In our case, age does not impact on the perceived usefulness or ease of use, but on the intention to use. This could be explained by the fact that, the technology under consideration, i.e., a smartphone app, is widespread; therefore, usage patterns and usefulness are shared by a large segment of the population. In addition, the sample we considered was of working age (see Table 1), and the questionnaire was disseminated via links, so the participants had enough technical skills to complete the questionnaire via computer or a smartphone. Intention to use could be influenced by age because, although the use of smartphone apps to achieve different goals is quite widespread among the population, the use of apps to promote well-being and manage stress may be concentrated among younger people, who tend to use their smartphones more during the day and are more familiar with app stores and installation methods [88,89]. 

## 5. Conclusions

In summary, the current study contributes to the scientific literature by deepening the comprehension of what personal and organizational variables may influence workers’ intentions to use new technologies, such as smartphone apps for stress management and well-being promotion. Specifically, this study underlines how the perception of self-efficacy in the use of smartphones and personal innovativeness could positively influence the perception of ease of use and usefulness of the technology examined and, consequently, positively affects the intention to use it in the future. In addition, from another perspective, the perceived organizational support for innovation contributes positively to the workers’ perceived ease of use and usefulness of new technologies, including smartphone apps. Moreover, it increases the intention to use it for well-being promotion and stress management. From a practical point of view, these findings could offer some critical hints to organizations and HRM. 

Organizations are always looking for new approaches to managing work-related stress. On the one hand, they are interested in saving resources, including financial resources, which are increasingly scarce. On the other hand, employers desire to increase the number of participants who benefit from such interventions. Indeed, the greater the number of participants in work-related stress management and well-being promotion interventions, the greater the benefits, both in terms of the mental and physical health if the employees, as well as employee satisfaction and improved productivity/work performance. 

Technologies could help us reach a broad number of workers at the same time. Moreover, using a smartphone for delivering stress management interventions can guarantee worker anonymity. However, not all employees agree on the use of new technologies to participate in well-being promotion interventions. For this reason, the organization needs to investigate technology acceptance, in terms of perceived usefulness and ease of use, before introducing these tools. This would help an organization decide what is beneficial; it simplifies any introduced or planned new technology to clarify (to employees) the convenience of adopting it. It is still useful for app developers to design apps, especially when they are to be placed in working contexts, considering the dimensions of usefulness and ease of use, making the advantages of using these apps as clear as possible, and simplifying the various steps leading to the purpose for which the apps are proposed/introduced.

It may also be convenient to investigate some variables involved in this process, such as those identified by this study, namely the perception of self-efficacy in using technology (in our case, smartphones) and personal innovativeness. It can be helpful to identify people within one’s own company that exhibit these characteristics, supporting workers who are less innovative or less competent in the use of technology. It is also relevant to investigate how employees perceive the organization from the point of view of organizational support for innovation. Our findings show that a higher support perception is linked to higher perceived usefulness and ease of use, which increases the likelihood of employees adopting new technologies. The reason is that if employees perceive that they are in an innovation-oriented company, they can better appreciate the value of innovation and be more confident that they will receive help (i.e., in learning how to use the technology). It is helpful because, if workers have low perceptions of support, employers can work on communication plans focused on technological innovations, their benefits, and emphasize the willingness to support employees when introducing new tools. Finally, few studies investigated the TAM in an Italian working context (see [90]). To the best of our knowledge, no studies have investigated the intention to use apps for the promotion of well-being in such contexts. Therefore, we can say that our research allowed us to test the validity of the acceptance model in the Italian workplace context and pave the way for new research that will facilitate the introduction of these new technologies, to ensure a higher level of well-being for workers.

We can also identify some limitations of the current study. Firstly, the study’s design was cross-sectional, which did not allow us to investigate the relationships between the variables in the long-term. In addition, the sample, although adequate for the statistical analysis carried out, was small and limited to the white-collar sector, which did not allow us to generalize the study to a broader section of the population. Finally, the study investigated the intention to use a hypothetical app without the possibility of examining the actual use of such a tool.

Future directions include designing another study with a longitudinal research design to identify causal relationships between variables, proposing a functioning app to workers to take into account objective data related to the actual use, and extending the research to a more significant number of participants, from different companies and covering different roles.

Smartphones are very popular in the general population and with workers, and smartphone apps are relatively easy to program and customize. Several people can use them simultaneously; they can guarantee the anonymity of participants, and apps can be used at any time. For this reason, they could be appropriate tools used to increase the activities implemented by the organization, to promote the well-being and health of the employees. However, to enhance the possibility of success of such a digital intervention, it is crucial to investigate the variables that could influence the intention to use such technologies among employees.

## Figures and Tables

**Table 1:** Descriptive Statistics.

Variables	1	2	3	4	5	6	7	9
1. Perceived Usefulness	(0.91)							
2. Perceived Ease Of Use	0.46 **	(0.92)						
3. Intention to use	0.72 **	0.50 **	(0.86)					
4. Specific Self-Efficacy	0.16 *	0.40 **	0.16 *	(0.94)				
5. Organizational Support for Innovation	0.30 **	0.36 **	0.32 **	0.19 **	(0.93)			
6. Personal Innovativeness with Technology	0.1 *	0.26 **	0.22 **	0.25 **	0.21 **	(0.83)		
7. Gender	−0.05	0.02	0.07	−0.08	0.004	−0.19 **	--	
8. Age	0.09	−0.13 *	−0.14 *	0.05	0.01	−0.11	−0.16 *	--
M	2.81	3.61	2.96	51.17	2.19	4.98	0.61	39.90
DS	0.73	0.69	0.81	16.60	0.77	1.27	0.49	9.45

Note: Cronbach’s on the diagonal. ** *p* < 0.01; * *p* < 0.05. Gender: male = 0, female = 1.

**Table 2:** Model Indices.

Models	χ^2^	*df*	CFI	TLI	RMSEA	SRMR	AIC	Comparison	Δχ^2^
M1	819.66 ***	417	0.93	0.92	0.06 (0.06, 0.07)	0.06	18,882.91		
M2	717.63 ***	412	0.95	0.94	0.05 (0.05 0.06)	0.05	18,788.87	M_1_ − M_2_	102.03 ***

Note: *** *p* < 0.001.

**Table 3:** Bootstrapping indirect effects.

**Indirect Effects** **Org Support → Intention**	**Est.**	**SE**	***p***	**CI 95%**
ORG SUP → PU → INT	0.12	0.04	0.005	(0.04, 0.20)
ORG SUP → PEOU → INT	0.03	0.02	0.074	(−0.03, 0.07)
ORG SUP → PEOU → PU → INT	0.04	0.02	0.009	(0.01, 0.07)
**Indirect Effects** **Smartphone Self-Efficacy → Intention**	**Est.**	**SE**	***p***	**CI 95%**
SSE → PEOU → INT	0.05	0.03	0.043	(0.02, 0.10)
SSE → PEOU → PU → INT	0.07	0.02	0.001	(0.03, 0.11)

Note: all parameter estimates are presented as standardized coefficients. Estimates (Est.). Standard Error (SE). Confidence interval (CI). Organizational Support for Innovation (ORG SUP). Perceived Usefulness (PU). Perceived Ease of Use (PEOU). Intention to Use (INT). Smartphone self-efficacy (SSE).

## Data Availability

All scales used for the study are available from the corresponding author upon reasonable request.
